# The Respiratory Commensal Bacterium *Corynebacterium pseudodiphtheriticum* as a Mucosal Adjuvant for Nasal Vaccines

**DOI:** 10.3390/vaccines11030611

**Published:** 2023-03-08

**Authors:** Ramiro Ortiz Moyano, Fernanda Raya Tonetti, Kohtaro Fukuyama, Mariano Elean, Mikado Tomokiyo, Yoshihito Suda, Vyacheslav Melnikov, Haruki Kitazawa, Julio Villena

**Affiliations:** 1 Laboratory of Immunobiotechnology, Reference Centre for Lactobacilli (CERELA-CONICET), Tucumán 4000, Argentina; rortiz@cerela.org.ar (R.O.M.); frayatonetti@gmail.com (F.R.T.); melean@cerela.org.ar (M.E.); 2 Food and Feed Immunology Group, Laboratory of Animal Food Function, Graduate School of Agricultural Science, Tohoku University, Sendai 981-8555, Japan; kotaro.fukuyama.p8@dc.tohoku.ac.jp (K.F.); mikado.tomokiyo.t4@dc.tohoku.ac.jp (M.T.); 3 Livestock Immunology Unit, International Education and Research Center for Food and Agricultural Immunology (CFAI), Graduate School of Agricultural Science, Tohoku University, Sendai 981-8555, Japan; 4 Department of Food, Agriculture and Environment, Miyagi University, Sendai 980-8572, Japan; suda@myu.ac.jp; 5 Gabrichevsky Research Institute for Epidemiology and Microbiology, 125212 Moscow, Russia; slavawho1@gmail.com

**Keywords:** *Corynebacterium pseudodiphtheriticum*, bacterium-like particles, mucosal adjuvant, respiratory infection, alveolar macrophages

## Abstract

Previously, we demonstrated that nasally administered *Corynebacterium pseudodiphtheriticum* 090104 (Cp) or its bacterium-like particles (BLPs) increase the resistance of mice against bacterial and viral respiratory pathogens by modulating the innate immunity. In this work, we evaluated the ability of Cp and BLPs to stimulate alveolar macrophages, and to enhance the humoral immune response induced by a commercial vaccine against *Streptococcus pneumoniae*. In the first set of experiments, Cp or the BLPs were incubated with primary cultures of murine alveolar macrophages and the phagocytic activity, and the production of cytokines was evaluated. The results revealed that Cp and BLPs were efficiently phagocyted by respiratory macrophages and that both treatments triggered the production of TNF-α, IFN-γ, IL-6, and IL-1β. In the second set of experiments, 3-week-old Swiss mice were intranasally immunized at days 0, 14, and 28 with the pneumococcal vaccine Prevenar^®^13 (PCV), Cp + PCV, or BLPs + PCV. On day 33, samples of bronco-alveolar lavages (BAL) and serum were collected for the study of specific antibodies. In addition, immunized mice were challenged with *S. pneumoniae* serotypes 6B or 19F on day 33 and sacrificed on day 35 (day 2 post-infection) to evaluate the resistance to the infection. Both Cp + PCV and BLPs + PCV groups had higher specific serum IgG and BAL IgA antibodies than the PCV control mice. In addition, the mice that were immunized with Cp + PCV or BLPs + PCV had lower lung and blood pneumococcal cell counts as well as lower levels of BAL albumin and LDH, indicating a reduced lung damage compared to the control mice. Improved levels of anti-pneumococcal antibodies were also detected in the serum and BAL samples after the challenges with the pathogens. The results demonstrated that *C. pseudodiphtheriticum* 090104 and its bacterium-like particles are capable of stimulating the respiratory innate immune system serving as adjuvants to potentiate the adaptive humoral immune response. Our study is a step forward in the positioning of this respiratory commensal bacterium as a promising mucosal adjuvant for vaccine formulations aimed at combating respiratory infectious diseases.

## 1. Introduction

Respiratory infections caused by viruses and bacteria spread throughout the world every year, severely affecting high-risk populations such as the elderly, infants, and immunocompromised patients. Individuals that are vaccinated against respiratory pathogens can prevent the severe pathological conditions caused by the infectious challenges. However, intramuscular and subcutaneous inoculations with vaccine antigens usually do not prevent the infections themselves [1]. Even in vaccinated subjects, pathogens can replicate in the respiratory tract, producing symptoms and spreading to other individuals. Then, nasally administered vaccines that mimic natural infections and generate both systemic and mucosal immunity can be useful tools to avoid severe cases of respiratory infections but also to prevent the spread of pathogens. Of note, most of the candidate antigens for the development of mucosal vaccines against respiratory infections have low antigenicity, and therefore, it is necessary to administer them together with adjuvants to induce protective immunity [2]. The lack of development of safe and effective mucosal adjuvants has limited the application of intranasal vaccines and therefore, only a few nasal vaccines are currently approved for humans, and all of them are live attenuated influenza vaccines [2].

*Corynebacterium pseudodiphtheriticum* is a commensal bacterium normally found in the human nasopharynx mucosa [3,4]. We demonstrated that the nasal administration of viable *C. pseudodiphtheriticum* 090104 to mice has a remarkable capacity to beneficially modulate the respiratory immune system, increasing the resistance to respiratory syncytial virus [5], *Streptococcus pneumoniae* [6], and hypermucoviscous carbapenemase-producing *Klebsiella pneumoniae* [7]. The protective effect of the 090104 strain against the different respiratory pathogens was related to its capacity to modulate the respiratory innate immune responses. In fact, our studies showed that *C. pseudodiphtheriticum* increased the expression of MHC-II molecules in CD11c^+^CD11b^low^CD103^+^ and CD11c^+^CD11b^high^CD103^−^ respiratory dendritic cells (DCs) [5], and stimulated CD45^+^SiglecF^+^ alveolar macrophages thus improving their production of interferon (IFN)-β and IFN-γ when Toll-like receptor 3 (TLR3) [5] or TLR2 [6] signaling pathways are activated. Therefore, we hypothesize that nasal priming with *C. pseudodiphtheriticum* 090104 modulates the activity of respiratory antigen-presenting cells (APCs), modifying their cytokine profile, which differentially coordinates innate immune mechanisms in the respiratory tract. Considering that the modulation of the activity of APCs may not only impact the innate immunity but also the adaptive immune responses, it is possible to speculate that *C. pseudodiphtheriticum* 090104 could also modulate antigen-specific humoral and cellular immune responses in the respiratory tract. However, this effect has not been evaluated so far.

Studies have demonstrated that non-pathogenic commensal microorganisms can be effectively used as adjuvants and delivery systems in the development of mucosal vaccines. Most of the research in this regard focused on genetically modified lactic acid bacteria (LAB) strains expressing antigens from bacterial and viral pathogens [8,9,10]. The oral or nasal administration of recombinant LAB have been shown to efficiently stimulate antigen-specific immune responses, both at the systemic and mucosal levels. However, the use of recombinant microorganisms in human vaccination raises concerns related to the potential for antibiotic and/or virulence factor gene dissemination into the environment. An interesting option for the replacement of genetically modified lactobacilli and lactococci was the development of bacterium-like particles (BLPs) obtained by the heat and acid treatments of LAB [11,12]. The BLPs from LAB strains do not contain cytoplasmic proteins or genetic material but preserve the cell-wall structure of the microorganisms, exposing the peptidoglycan. Therefore, BLPs from LAB have an increased ability to bind proteins containing the lysine motifs (LysM) to the cell-wall peptidoglycan [11,12]. It was shown that recombinant antigens carrying the LysM had a significantly higher ability to induce systemic and mucosal-specific immune responses when they are attached to BLPs from LAB in comparison to being administered alone [13,14], indicating that BLPs can function not only as delivery systems but also as mucosal adjuvants.

The nasal priming with immunomodulatory beneficial microbes such as LAB improves the respiratory immune responses, increasing the protection against bacterial and viral pathogens (reviewed in [4]). Interestingly, it was shown that the viability of immunomodulatory LAB is not an essential requirement for them to exert their beneficial effects on the respiratory tract [15,16,17,18]. Then, the use of non-viable immunomodulatory microorganisms including BLPs is an interesting option to regulate immunity in the respiratory mucosa of an immunocompromised host since the viable bacteria may represent a potential threat. In this regard, we have obtained BLPs derived from *C. pseudodiphtheriticum* 090104 and we have evaluated their capacity to modulate respiratory immune responses [5,6]. In our hands, BLPs from *C. pseudodiphtheriticum* 090104 administered nasally to infant mice were as effective as the viable respiratory commensal bacterium at modulating the activation of respiratory DCs and alveolar macrophages [5,6]. These results indicate that BLPs from *C. pseudodiphtheriticum* 090104 could be an alternative of great interest for the development of mucosal vaccines for the respiratory tract if their adjuvant properties are demonstrated and studied in depth.

Considering this background, in this work we evaluated the ability of *C. pseudodiphtheriticum* 090104 and its BLPs to stimulate alveolar macrophages and to enhance the humoral immune response induced by a commercial vaccine against *S. pneumoniae*. To the best of our knowledge, there are no reports about the capacity of respiratory commensal bacteria or their non-living cells as mucosal adjuvants for nasal vaccine development.

## 2. Materials and Methods

### 2.1. C. pseudodiphtheriticum and Obtention of BLPs

*C. pseudodiphtheriticum* 090104 was cultured at 37 °C for 18 h (late log phase) in trypticase soy broth. Bacteria suspensions were prepared as previously described [5,6]. For the experiments, the 090104 strain was washed with sterile 0.01 M phosphate-buffered saline (PBS, pH 7.2) at 3000× *g* for 10 min and suspended in sterile PBS. The heat–chemical treatment of the respiratory commensal bacterium to generate BLPs was performed as previously described [5,6]. Briefly, *C. pseudodiphtheriticum* 090104 from a fresh overnight culture was washed once with sterile distilled water at 13,000× *g* for 10 min. The pellet was suspended in 20 mL of 0.1 M HCl and boiled in a water bath for 45 min. The obtained BLPs were washed three times (PBS, pH 7.2). Finally, BLPs were resuspended in PBS and stored at −20 °C. The number of BLPs per milliliter was adjusted according to the CFU/mL determined in the starting culture. Viability of BLPs was checked by plating the suspensions and several dilutions on BHI agar plates and broth.

### 2.2. Electron Microscopy

Scanning (SEM) and transmission (TEM) electron microscopies were performed in the Centro de Investigaciones y Servicios de Microscopía Electronica (CISME–CONICET, Tucuman, Argentina). Samples were prepared and processed according to standard procedures. Briefly, *C. pseudodiphtheriticum* 090104 and its BLPs were fixed with Karnovsky fixative for 24 h at 4 °C. Then, samples were washed with 0.1 M sodium phosphate buffer (pH 7.4) and post-fixed in a solution of 1% osmium tetroxide in sodium phosphate buffer for 16 h. Dehydratation was produced by graded ethanol series, and then samples were embedded in Spurr resin. Ultrathin sections were cut with an ultramicrotome and examined with a Zeiss Libra 120 microscope for TEM and a Supra55VP microscope for SEM.

### 2.3. Modulation of Alveolar Macrophages by C. pseudodiphtheriticum and Its BLPs

Murine alveolar macrophages were used for the experiments. Murine alveolar macrophage primary cultures were performed, as described previously [17,18]. Macrophages were obtained from infant mice via broncho-alveolar (BAL) samples. BAL cells were cultured in RPMI 1640 medium containing 100 U/mL penicillin–streptomycin, 1 mM L-glutamine, and 10% FBS and were seeded in 24-well plates (10^5^ cells/well). After an incubation for 2 h at 37 °C at 5% CO_2_ to promote adherence, non-adherent cells were washed, and macrophages were recovered in RPMI 1640 medium with 10% FBS, 1 mM L-glutamine, and 100 U/mL penicillin/streptomycin at 37 °C at 5% CO_2_. For light microscopy analysis, murine alveolar macrophages (1.0 × 10^6^ cells/well) were seeded in wells of glass-covered 6-well plates and cultured for 16 h at 37 °C at 5% CO_2_. Cells were stimulated with viable *C. pseudodiphtheriticum* or its BLPs (1.0 × 10^8^ cells/mL) for 30 min. Samples were fixed with methanol and stained with Giemsa stain (Wako). Phagocytosis was observed by light microscope to determine the number of bacteria/BLPs in each macrophage. Supernatants were collected 24 h after stimulation for cytokines analysis with commercially available ELISA technique kits as described below.

### 2.4. Measurement of Cytokines’ Production by Alveolar Macrophages

For the determination of cytokines’ production by murine alveolar macrophages, ELISAs were performed. Interleukin (IL)-6 (Mouse IL-6 Quantikine ELISA Kit), IL-1β (Mouse IL-1 beta/IL-1F2 DuoSet ELISA Kit), interferon (IFN)-γ (Mouse IFN-gamma Quantikine ELISA Kit), and tumor necrosis factor (TNF)-α (Mouse TNF-α 236 ELISA Kit) concentrations were measured with commercially ELISA technique kits following the manufacturer’s recommendations (R&D Systems, Minneapolis, MN, USA).

### 2.5. Animal Experiments

Three-week-old male Swiss albino mice were obtained from the closed colony at CERELA (San Miguel de Tucumán, Argentina). Mice were housed in plastic cages and environmental conditions were kept constant, in agreement with the standards for animal housing. Experiments with animals were performed in accordance with the guide for the care and use of laboratory animals and were approved by the CERELA-CONICET Animal Care and Ethics Committee under the BIOT-CRL/19 protocol.

The dose of adjuvant for the experiments in this study was selected according to our previous studies evaluating the immunomodulatory effects of *C. pseudodiphtheriticum*, which demonstrated that the optimal dose to induce immunomodulation was 10^8^ CFU [5,6]. Then, for immunization experiments, different groups of mice received 10 ug of the pneumococcal vaccine Prevenar^®^13 (PCV), PCV together with 10^8^ cells of *C. pseudodiphtheriticum* 090104 or PCV and 10^8^ BLPs by the nasal route on days 0, 14, and 28. Animals received 25 μL of PBS containing the different vaccine formulations by administering 12.5 μL into each nostril. Five days after the last immunization (day 33), serum and BAL samples were taken for the determination of specific antibodies. 

### 2.6. Determination of Antibodies

IgA and IgG-specific anti-PCV antibodies were measured by ELISA. One μg of PCV per well was used to coat plates overnight at 4 °C. Plates were then blocked with albumin. Appropriate dilutions of the samples (serum 1:20; BAL 1:2) were incubated for 1 h at 37 °C. Peroxidase conjugated anti-mouse IgG, or IgA antibodies (1:500) (Sigma-Aldrich, St. Louis, MO, USA) were added and incubated for 1 h at 37 °C. TMB substrate reagent (Sigma-Aldrich) was used to reveal the reaction. The concentration of antibodies was measured with reference to standard curves using known amounts of the respective mouse immunoglobulin (Sigma-Aldrich).

### 2.7. Infection Challenge Experiments

*S. pneumoniae* serotypes 6B and 19F were grown on blood agar at 37 °C for 18 h. Todd Hewitt broth (THB, Oxoid, Cambridge, UK) was used to obtain pneumococci for challenge experiments. Grown colonies were suspended in THB and incubated overnight at 37 °C. *S. pneumoniae* was harvested by centrifugation at 3600× *g* for 10 min, and then washed with sterile PBS. Mice were infected nasally with 10^6^ CFU of pneumococci per mouse. Lung and blood pneumococcal cell counts were determined as described before [6,19]. In addition, albumin content and lactate dehydrogenase (LDH) activity were determined in BAL of infected animals, as described previously [6,19]. These parameters allow us to indirectly measure increased permeability of the bronchoalveolar–capillarity barrier and general cytotoxicity, respectively.

### 2.8. Statistical Analysis

Each experimental group consisted of 4 mice per group at each time point and experiments were performed in triplicates (*n* = 12 for each parameter studied). Results were expressed as mean ± standard deviation (SD). The differences between groups were analyzed using student t-test. Differences were considered significant at *p* < 0.05. ANOVA one-way was used for analysis of variance among multiple groups.

## 3. Results

### 3.1. Obtention of BLPs from the Respiratory Commensal C. pseudodiphtheriticum

**Figure 1:**
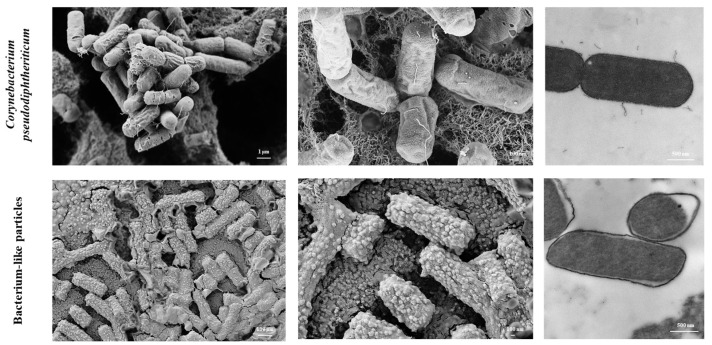
Scanning (SEM) and transmission (TEM) electron microscopy analysis. *Corynebacterium pseudodiphtheriticum* 090104 untreated cells and bacterium-like particles (BLPs) obtained from the 090104 strain by heat–acid treatment. Ultrathin sections cuts were examined with a Zeiss libra 120 and a Supra55VP microscopes for TEM and SEM, respectively.

The heat–acid treatment of *C. pseudodiphtheriticum* 090104 resulted in non-living BLPs particles. The lack of viability from the BLPs derived from the 090104 strain was confirmed by cultures in the BHI broth and agar. The TEM analysis revealed that this procedure partially released or degraded the DNA and proteins from the BLPs (Figure 1). BLPs from *C. pseudodiphtheriticum* 090104 preserved the same size and shape of the respiratory commensal bacterium, as shown by both the SEM and TEM analysis (Figure 1). Peptidoglycan, the main component of the Gram-positive bacteria’s cell wall, was exposed by the harsh acid treatment (Figure 1).

### 3.2. C. pseudodiphtheriticum and Its BLPs Modulate Alveolar Macrophages’ Function

**Figure 2:**
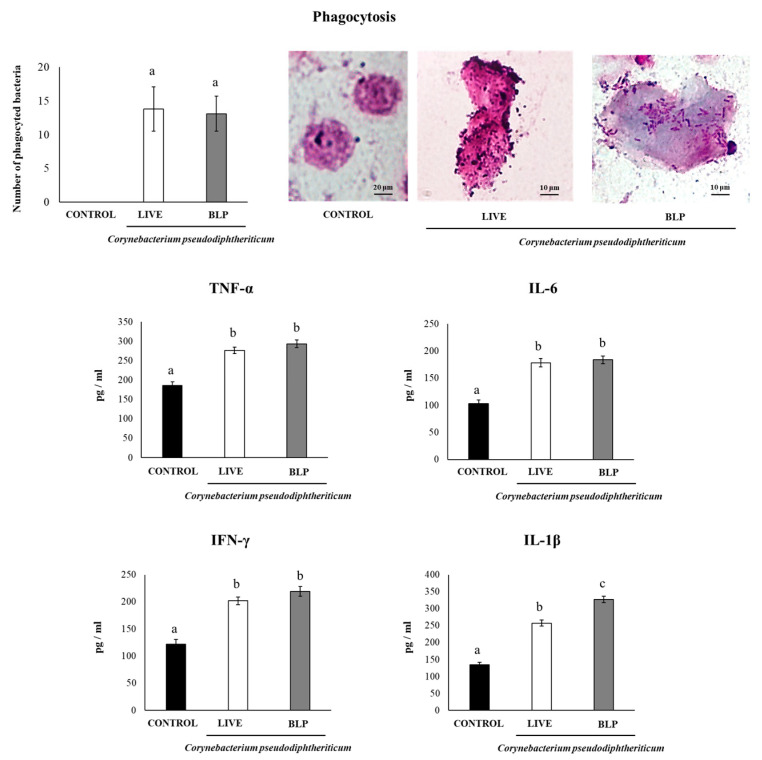
Interaction of *Corynebacterium pseudodiphtheriticum* 090104 and its bacterium-like particles (BLPs) with murine alveolar macrophages. Phagocytosis of *C. pseudodiphtheriticum* and its BLPs by murine alveolar macrophages was evaluated by microscopic analysis. The production of TNF-α, IL-1β, IL-6, and IFN-γ in the culture supernatants of alveolar macrophages stimulated with bacteria or BLPs was evaluated after 24 h. Untreated murine alveolar macrophages were used as controls. Results represent data from three independent experiments (*n* = 12) and are expressed as mean ± standard deviation. Letters indicate significant differences between the groups (*p* < 0.05).

We evaluated the capacity of *C. pseudodiphtheriticum* and its BLPs to be phagocyted by alveolar macrophages and to induce their activation. For these experiments, we performed primary cultures of murine alveolar macrophages. These respiratory immune cells were incubated with *C. pseudodiphtheriticum* or its BLPs and the numbers of bacteria/BLPs were counted in macrophages (Figure 2). An average of 14 bacteria/BLPs was detected in murine alveolar macrophages. No significant differences were found when *C. pseudodiphtheriticum*-treated macrophages were compared with those stimulated with BLPs, indicating that both are equally phagocyted. In addition, we determined the levels of TNF-α, IL-1β, IL-6, and IFN-γ in the supernatants of the primary cultures of murine alveolar macrophages (Figure 2). Both *C. pseudodiphtheriticum* and its BLPs significantly increased the levels of the four cytokines when compared to non-stimulated macrophages. No differences between the treatments were found when the levels of TNF-α, IL-6, and IFN-γ were analyzed. However, the concentrations of IL-1β were significantly higher in BLP-treated alveolar macrophages than in the cells stimulated with *C. pseudodiphtheriticum* (Figure 2).

### 3.3. C. pseudodiphtheriticum and Its BLPs Improve the Immune Response to a Pneumococcal Vaccine

**Figure 3:**
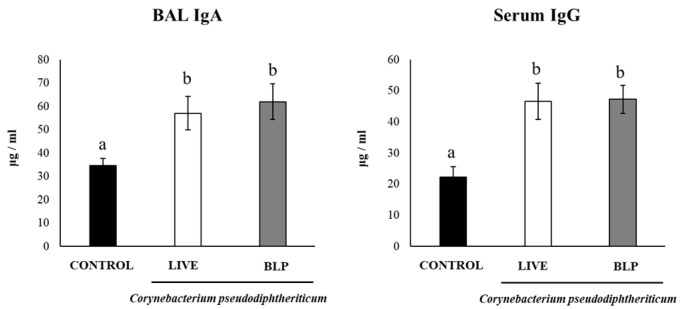
Humoral immune response induced by the immunization with the pneumococcal conjugate vaccine (PCV) plus *Corynebacterium pseudodiphtheriticum* 090104 or its bacterium-like particles (BLPs) in infant mice. Swiss albino infant mice (3 weeks old) were immunized by the nasal route with PCV or PCV plus *C. pseudodiphtheriticum* 090104 or it BLPs on days 0, 14, and 28. Five days after the last immunization (day 33), serum and broncho-alveolar lavage (BAL) samples were obtained for the determination of specific antibodies. Concentration of PCV-specific IgA in BAL and IgG in serum were determined with ELISA. Results represent data from three independent experiments (*n* = 6) and are expressed as mean ± standard deviation. Letters indicate significant differences between the groups (*p* < 0.05).

We next evaluated whether *C. pseudodiphtheriticum* and its BLPs were able to enhance the humoral immune response induced by a nasally administered pneumococcal vaccine. For this purpose, infant Swiss mice (3 weeks old) were intranasally immunized at days 0, 14, and 28 with the pneumococcal conjugate vaccine Prevenar^®^13 (PCV), and the pneumococcal vaccine plus *C. pseudodiphtheriticum* or its BLPs. On day 33, samples of blood and BAL were obtained for the evaluation of specific antibodies. The immunization of mice with PCV by the nasal route induced the production of specific BAL IgA and serum IgG (Figure 3). Of note, when the PCV was administered with *C. pseudodiphtheriticum* or its BLPs, higher levels of anti-pneumococcal IgA and IgG were found. No significant differences were detected between the groups of mice immunized with PCV plus *C. pseudodiphtheriticum* or its BLPs (Figure 3).

**Figure 4:**
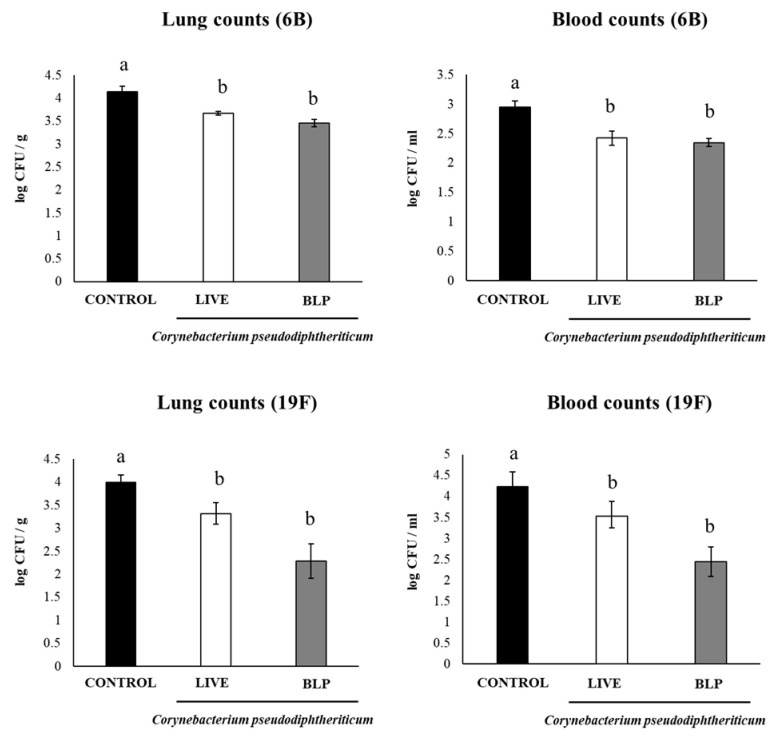
Resistance to *Streptococcus pneumoniae* infection induced by the immunization with the pneumococcal conjugate vaccine (PCV) plus *Corynebacterium pseudodiphtheriticum* 090104 or its bacterium-like particles (BLPs) in infant mice. Swiss albino infant mice (3 weeks old) were immunized by the nasal route with PCV or PCV plus *C. pseudodiphtheriticum* 090104 or it BLPs on days 0, 14, and 28. Five days after the last immunization (day 33), mice were nasally challenge with *S. pneumoniae* serotypes 6B or 19F. Lung and blood pneumococcal cell counts were performed on day 2 post-infection. Results represent data from three independent experiments (*n* = 12) and are expressed as mean ± standard deviation. Letters indicate significant differences between the groups (*p* < 0.05).

**Figure 5:**
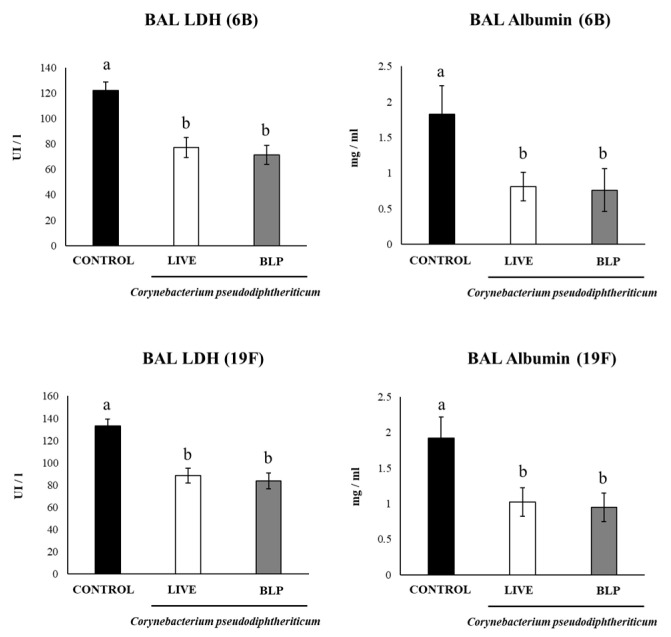
Resistance to *Streptococcus pneumoniae* infection induced by the immunization with the pneumococcal conjugate vaccine (PCV) plus *Corynebacterium pseudodiphtheriticum* 090104 or its bacterium-like particles (BLPs) in infant mice. Swiss albino infant mice (3 weeks old) were immunized by the nasal route with PCV or PCV plus *C. pseudodiphtheriticum* 090104 or it BLPs on days 0, 14, and 28. Five days after the last immunization (day 33), mice were nasally challenge with *S. pneumoniae* serotypes 6B or 19F. The determination of albumin and lactate dehydrogenase (LDH) in broncho-alveolar lavage (BAL) samples were performed on day 2 post-infection. Results represent data from three independent experiments and are expressed as mean ± standard deviation. Letters indicate significant differences between the groups (*p* < 0.05).

In addition, to evaluate the protective effect of the immunization protocols including *C. pseudodiphtheriticum* and its BLPs, immunized mice were challenged with *S. pneumoniae* serotypes 6B or 19F on day 33 and sacrificed on day 35 (day 2 post-infection). The groups of mice receiving the PCV plus with *C. pseudodiphtheriticum* or its BLPs had significantly lower lung and blood pneumococcal cell counts than the controls, even for the most virulent serotype 19F (Figure 4). We also detected lower levels of BAL albumin and LDH in mice treated with the PCV plus *C. pseudodiphtheriticum* or its BLPs than the controls, indicating a reduced lung damage (Figure 5). 

**Figure 6:**
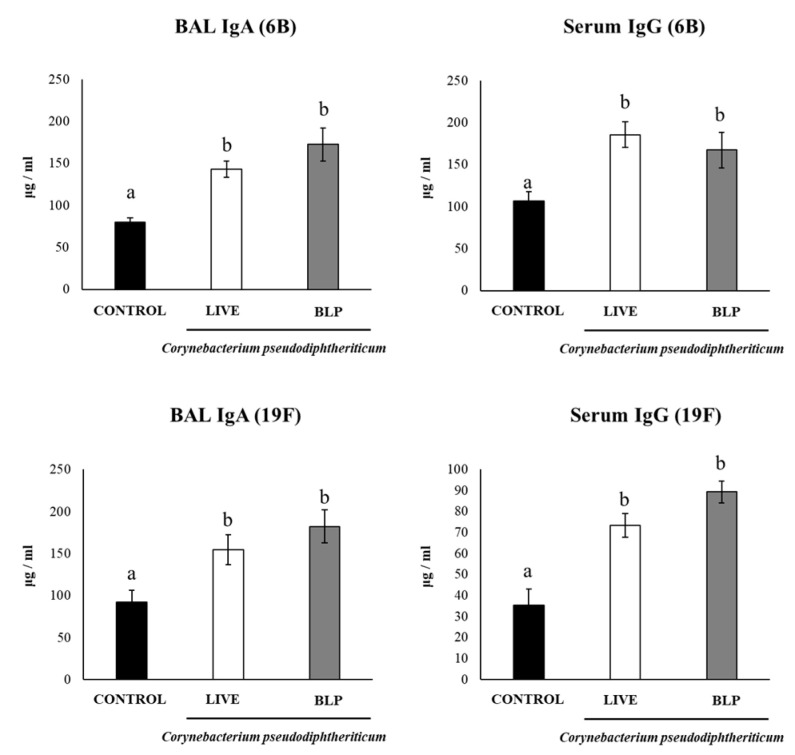
Resistance to *Streptococcus pneumoniae* infection induced by the immunization with the pneumococcal conjugate vaccine (PCV) plus *Corynebacterium pseudodiphtheriticum* 090104 or its bacterium-like particles (BLPs) in infant mice. Swiss albino infant mice (3 weeks old) were immunized by the nasal route with PCV or PCV plus *C. pseudodiphtheriticum* 090104 or it BLPs on days 0, 14, and 28. Five days after the last immunization (day 33), mice were nasally challenge with *S. pneumoniae* serotypes 6B or 19F. The determination of anti-pneumococcal IgA in broncho-alveolar lavage (BAL) samples and IgG in serum were performed on day 2 post-infection. Results represent data from three independent experiments (*n* = 12) and are expressed as mean ± standard deviation. Letters indicate significant differences between the groups (*p* < 0.05).

Finally, we determined the levels of BAL IgA and serum IgG after the challenge with the pneumococcal serotypes (Figure 6). As expected, the levels of respiratory and systemic anti-pneumococcal antibodies were significantly higher after the challenge with the pathogens (Figure 6) than after immunization (Figure 3) in all the experimental groups. However, the groups of mice receiving the PCV plus *C. pseudodiphtheriticum* or its BLPs had significantly higher levels of BAL IgA and serum IgG antibodies than the controls. No significant differences were found when comparing *C. pseudodiphtheriticum* with BLPs in the improvement of the humoral immune response (Figure 6).

## 4. Discussion

In recent decades, great efforts have been made to find mucosal adjuvants for the generation of nasal vaccines. Advances in the understanding of the important role of APCs and pattern recognition receptors (PRRs) in the link between innate and adaptive immunity have focused adjuvant investigations on a new direction. The activation of the innate immune system via PRRs in general, and TLRs in particular, was shown to be of great importance for inducing lasting adaptive immunity. The activation of TLRs in APCs can modulate the development of both humoral and cellular adaptive immune responses, making the TLRs expressed in APCs attractive targets for the development of vaccine adjuvants [20]. In this regard, it was reported that the nasal administration of antigens from respiratory pathogens together with the TLR4 agonist monophosphoryl lipid A stimulated a specific Th1 response and IgA production in the respiratory tract [21,22]. The synthetic analog of double-stranded RNA and TLR3 agonist poly (I:C) [23,24], and the TLR9 agonist unmethylated CpG oligodeoxynucleotide [25] were also shown to enhance the activity of APCs and improve IgA production in the respiratory mucosa. On the other hand, it was reported that the TLR5 agonist flagellin is capable of enhancing the mucosal IgA production when nasally administered with vaccine antigens [19,26]. Immunomodulatory LAB have been extensively studied as safe and effective mucosal adjuvants and antigen delivery vehicles for mucosal vaccine development [27,28]. These Gram-positive microorganisms are generally recognized as safe (GRAS) bacteria and their cell walls as well as the molecules exposed on their surface such as peptidoglycan, teichoic acid, lipoteichoic acid, and lipopeptides have been characterized as efficient activators of PRRs such as TLR2, TLR6, NOD1, and NOD2 expressed in APCs [27,28]. The nasal administration of LAB or their cellular fractions together with antigens have been shown to generate mixed Th1 and Th2 responses in the respiratory tract and enhanced levels of specific IgA [19,29,30], indicating that Gram-positive GRAS microorganisms can be a valuable tool in the development of mucosal vaccines. 

The advances in the knowledge of the composition and functions of the microbiota associated with the respiratory tract have made it possible to consider some Gram-positive respiratory commensal bacteria as immunomodulatory microorganisms with positive effects on health [3,4]. In this regard, we showed that the human nasopharynx *C. pseudodiphtheriticum* 090104 strain improved MHC-II expression in CD11c^+^CD11b^low^CD103^+^ and CD11c^+^CD11b^high^CD103^−^ lung DCs and CD45^+^SiglecF^+^ alveolar macrophages [5,6], indicating its ability to stimulate APCs. Furthermore, the nasal administration of *C. pseudodiphtheriticum* 090104 to mice enhanced the capacity of alveolar macrophages to produce IFN-β and IFN-γ after the challenge with TLR2 agonists [6]. In this work, we deepen and extend these findings by demonstrating that *C. pseudodiphtheriticum* 090104 is efficiently phagocyted by alveolar macrophages in vitro, and that this commensal bacterium induces the production of TNF-α, IL-1β, IL-6, and IFN-γ in this APC population. Antigen presentation in the respiratory tract is mediated by DCs and macrophages, which constitutively express MHC-I or MHC-II and have a high expression of co-stimulatory molecules, such as CD80 and CD86 [31,32]. Respiratory APCs engulf pathogens and present their peptides to naïve CD8^+^ or CD4^+^ T cells providing protection from pathogen infections [32]. Alveolar macrophages have been suggested to play a functional role in antigen presentation during tuberculosis and *Cryptococcus neoformans* infection in humans [33,34]. In contrast, studies in mice models have demonstrated that alveolar macrophages are poor APCs compared to DCs and other macrophages of the respiratory tract [31]. However, the role of alveolar macrophages in the generation of both humoral and cellular adaptive responses in the respiratory tract should not be underestimated. Alveolar macrophages are located on the luminal surface of the respiratory mucosa, and therefore, they are the first to encounter antigens and pathogens [35]. It was estimated that alveolar macrophages can handle up to 10 intratracheally injected bacteria before there is “spillover” of the bacteria to the lung DCs [36,37]. Interestingly, it was shown that alveolar macrophages can take pathogens before lung DCs, and that a fraction of pathogen-bearing alveolar macrophages migrate to draining the lymph nodes of the lung [38,39]. In addition, recent studies demonstrated the key role of alveolar macrophages in the process referred as cross-presentation [40], in which APCs uptake virus-infected cells and present their antigens to naive CD8^+^ T cells via MHC-I [41]. In a murine model of influenza infection, it was shown that alveolar macrophages are required for the expansion of antigen-specific CD8^+^ T cells and CD103^+^CD8^+^ resident memory T cells and the inhibition of influenza replication during a secondary infection [40]. It was also reported that the cytokines produced by alveolar macrophages after their encounter with pathogens can influence the degree of activity and maturation of neighboring DCs [31]. In fact, cytokines such as TNF-α, IL-1β, IL-6, and IFN-γ have been shown to significantly potentiate the activation of DCs [35,40,42]. Furthermore, alveolar macrophages highly express IL-18, which is of importance for the development of lung CD103^+^CD8^+^ resident memory T cells during viral infections [40]. 

Our previous [5,6] and present results have allowed us to speculate that nasally administered *C. pseudodiphtheriticum* 090104 could improve the activation of APCs including alveolar macrophages, beneficially impacting the generation of adaptive immune responses. In fact, the in vivo immunization studies performed here demonstrated the capacity of the 090104 strain to enhance both respiratory and systemic immune responses when administered with a pneumococcal vaccine. In our hands, the nasal administration of the PCV plus *C. pseudodiphtheriticum* significantly enhanced the production of specific BAL IgA and serum IgG. Both types of antibodies have been demonstrated to be crucial in the protection against pneumococcal infections. Mucosal surfaces are protected by secretory IgA (sIgA). These antibodies are locally produced by plasma cells in the lamina propria of the respiratory tract [43,44]. Antigen-specific IgA synthetized by B cells in cooperation with Th cells is released in the lamina propria and interacts with the polymeric Ig receptor (pIgR), which is expressed by respiratory epithelial cells to be transported to the airway [43,44]. Several works have demonstrated the important protective role of sIgA against pneumococcal infection. Early studies showed that sIgA directed to *S. pneumoniae* capsular polysaccharide improves the complement-mediated killing of the pathogen [45]. It was also reported that mice immunized with a mucosal vaccine that induced sIgA were protected against pneumococcal infection while the same treatment was not able to induce protection against the nasal challenge with *S. pneumoniae* in pIgR^−/−^ mice [46,47,48]. Furthermore, it was demonstrated that IgA^−/−^ mice immunized with a PspA-based vaccine were not able to eliminate pneumococcal carriage in the nasal cavity because of the lack of mucosal-specific antibodies [49]. In contrast, wild-type mice receiving the PspA-based vaccine had high levels of specific IgA in the respiratory tract and essentially no *S. pneumoniae* was detected in the nasal washes. On the other hand, as it has been described for serum neutralizing IgG antibodies against viral surface proteins, serum anti-pneumococcal antibodies of the IgG-type play a central role in controlling the spread of *S. pneumoniae* [45]. Dectin-2 deficiency, which impairs specific IgG production induced by a pneumococcal polysaccharide vaccine, significantly increase the susceptibility of mice to the *S. pneumoniae* infection [50]. Passive immunization of mice with specific IgG1 and IgG2 anti-pneumococcal antibodies resulted in the C3c deposition on bacterial surfaces and increased the resistance of animals to the challenge with the pathogen [51]. Then, the improved levels of specific BAL IgA and serum IgG antibodies induced by *C. pseudodiphtheriticum* when administered with the PCV can explain the higher resistance of mice against *S. pneumoniae* 6B and 19F as well as the reduced lung damage compared to mice immunized only with PCV.

Despite the positive results in terms of the beneficial modulation of the respiratory immunity obtained with *C. pseudodiphtheriticum* 090104 in our previous and present works, there is still concern about the use of viable bacteria of this species because of the reports indicating their capacity to induce infections in patients with underlying cardiac or pulmonary diseases [52,53] or in patients with mechanical ventilation, such as those with severe COVID-19 [54]. Then, BLP derived from *C. pseudodiphtheriticum* 090104 evaluated in this work can help to alleviate these concerns associated with live bacteria because they are non-living but retain the immunomodulatory properties. In fact, the BLPs were able to be phagocyted and stimulate cytokine production by alveolar macrophages, potentiating the humoral, mucosal, and systemic protective immune responses induced by the PCV as efficiently as the viable *C. pseudodiphtheriticum*. In addition, the immunization with PCV and BLP derived from *C. pseudodiphtheriticum* 090104 was as efficient as the viable respiratory commensal bacterium to protect mice against pneumococcal colonization and lung damage. Of note, it was reported that the nasal immunization of adult mice with ovalbumin plus LPS from *Alcaligenes faecalis* or cholera toxin isolated from *Vibrio cholerae* induced similar levels of specific respiratory IgA and serum IgG [55]. However, authors found differences between the vaccination treatments when specific cellular immune responses were evaluated. While both immunization treatments induced T cells to produce IL-17, only the cholera toxin stimulated the production of IFN-γ. Therefore, an important point for future research would be the evaluation of the specific cellular immunity induced by *C. pseudodiphtheriticum* 090104 and its BLPs to determine if they are equally effective or not.

## 5. Conclusions

Although nasal immunization is non-invasive, simpler, less painful, and enables self-administration, this type of vaccine needs safe and strong adjuvants to induce protective immunity. Then, successful nasal vaccination requires the development of appropriate mucosal adjuvants. Here, we demonstrated that the nasal immunization with PCV together with *C. pseudodiphtheriticum* or its BLPs elicited PCV-specific antibody responses in both respiratory and systemic compartments and improved the protection against pneumococcal infections. The results indicate that *C. pseudodiphtheriticum* and its BLPs should be further studied to position them as mucosal adjuvants for the development of efficient nasal vaccines to avoid the parenteral route and increase the patient’s compliance. BLPs would have advantages compared to live bacteria in terms of security.

## Data Availability

All the data related to this project are presented here.

## References

[1] Takaki H., Ichimiya S., Matsumoto M., Seya T. (2018). Mucosal Immune Response in Nasal-Associated Lymphoid Tissue upon Intranasal Administration by Adjuvants. J. Innate Immun..

[2] Xu H., Cai L., Hufnagel S., Cui Z. (2021). Intranasal Vaccine: Factors to Consider in Research and Development. Int. J. Pharm..

[3] de Steenhuijsen Piters W.A.A., Binkowska J., Bogaert D. (2020). Early Life Microbiota and Respiratory Tract Infections. Cell Host Microbe.

[4] Andrade B.G., Cuadrat R.R., Tonetti F.R., Kitazawa H., Villena J. (2022). The Role of Respiratory Microbiota in the Protection against Viral Diseases: Respiratory Commensal Bacteria as next-Generation Probiotics for COVID-19. Biosci. Microbiota Food Health.

[5] Kanmani P., Clua P., Vizoso-Pinto M.G., Rodriguez C., Alvarez S., Melnikov V., Takahashi H., Kitazawa H., Villena J. (2017). Respiratory Commensal Bacteria *Corynebacterium pseudodiphtheriticum* Improves Resistance of Infant Mice to Respiratory Syncytial Virus and *Streptococcus pneumoniae* Superinfection. Front. Microbiol..

[6] Ortiz Moyano R., Raya Tonetti F., Tomokiyo M., Kanmani P., Vizoso-Pinto M.G., Kim H., Quilodrán-Vega S., Melnikov V., Alvarez S., Takahashi H. (2020). The Ability of Respiratory Commensal Bacteria to Beneficially Modulate the Lung Innate Immune Response Is a Strain Dependent Characteristic. Microorganisms.

[7] Dentice Maidana S., Ortiz Moyano R., Vargas J.M., Fukuyama K., Kurata S., Melnikov V., Jure M.Á., Kitazawa H., Villena J. (2022). Respiratory Commensal Bacteria Increase Protection against Hypermucoviscous Carbapenem-Resistant *Klebsiella pneumoniae* ST25 Infection. Pathogens.

[8] Szatraj K., Szczepankowska A.K., Chmielewska-Jeznach M. (2017). Lactic Acid Bacteria—Promising Vaccine Vectors: Possibilities, Limitations, Doubts. J. Appl. Microbiol..

[9] Cho S.W., Yim J., Seo S.W. (2020). Engineering Tools for the Development of Recombinant Lactic Acid Bacteria. Biotechnol. J..

[10] Villena J., Kitazawa H. (2020). The Modulation of Mucosal Antiviral Immunity by Immunobiotics: Could They Offer Any Benefit in the SARS-CoV-2 Pandemic?. Front. Physiol..

[11] Audouy S.A.L., van Roosmalen M.L., Neef J., Kanninga R., Post E., van Deemter M., Metselaar H., van Selm S., Robillard G.T., Leenhouts K.J. (2006). *Lactococcus lactis* GEM Particles Displaying Pneumococcal Antigens Induce Local and Systemic Immune Responses Following Intranasal Immunization. Vaccine.

[12] Raya Tonetti F., Arce L., Salva S., Alvarez S., Takahashi H., Kitazawa H., Vizoso-Pinto M.G., Villena J. (2020). Immunomodulatory Properties of Bacterium-Like Particles Obtained From Immunobiotic Lactobacilli: Prospects for Their Use as Mucosal Adjuvants. Front. Immunol..

[13] Arce L.P., Raya Tonetti M.F., Raimondo M.P., Müller M.F., Salva S., Álvarez S., Baiker A., Villena J., Vizoso Pinto M.G. (2020). Oral Vaccination with Hepatitis E Virus Capsid Protein and Immunobiotic Bacterium-Like Particles Induce Intestinal and Systemic Immunity in Mice. Probiotics Antimicro. Prot..

[14] Raya-Tonetti F., Müller M., Sacur J., Kitazawa H., Villena J., Vizoso-Pinto M.G. (2021). Novel LysM Motifs for Antigen Display on Lactobacilli for Mucosal Immunization. Sci. Rep..

[15] Tomosada Y., Chiba E., Zelaya H., Takahashi T., Tsukida K., Kitazawa H., Alvarez S., Villena J. (2013). Nasally Administered *Lactobacillus rhamnosus* Strains Differentially Modulate Respiratory Antiviral Immune Responses and Induce Protection against Respiratory Syncytial Virus Infection. BMC Immunol..

[16] Clua P., Kanmani P., Zelaya H., Tada A., Kober A.K.M.H., Salva S., Alvarez S., Kitazawa H., Villena J. (2017). Peptidoglycan from Immunobiotic *Lactobacillus rhamnosus* Improves Resistance of Infant Mice to Respiratory Syncytial Viral Infection and Secondary Pneumococcal Pneumonia. Front. Immunol..

[17] Clua P., Tomokiyo M., Raya Tonetti F., Islam M.A., García Castillo V., Marcial G., Salva S., Alvarez S., Takahashi H., Kurata S. (2020). The Role of Alveolar Macrophages in the Improved Protection against Respiratory Syncytial Virus and Pneumococcal Superinfection Induced by the Peptidoglycan of *Lactobacillus rhamnosus* CRL1505. Cells.

[18] Raya Tonetti F., Clua P., Fukuyama K., Marcial G., Sacur J., Marranzino G., Tomokiyo M., Vizoso-Pinto G., Garcia-Cancino A., Kurata S. (2022). The Ability of Postimmunobiotics from *L. rhamnosus* CRL1505 to Protect against Respiratory Syncytial Virus and Pneumococcal Super-Infection Is a Strain-Dependent Characteristic. Microorganisms.

[19] Laiño J., Villena J., Suvorov A., Zelaya H., Moyano R.O., Salva S., Alvarez S. (2018). Nasal Immunization with Recombinant Chimeric Pneumococcal Protein and Cell Wall from Immunobiotic Bacteria Improve Resistance of Infant Mice to *Streptococcus pneumoniae* Infection. PLoS ONE.

[20] Xu M., Li N., Fan X., Zhou Y., Bi S., Shen A., Wang B. (2022). Differential Effects of Toll-Like Receptor Signaling on the Activation of Immune Responses in the Upper Respiratory Tract. Microbiol. Spectr..

[21] Baldridge J. (2000). Monophosphoryl Lipid A Enhances Mucosal and Systemic Immunity to Vaccine Antigens Following Intranasal Administration. Vaccine.

[22] Iwasaki T., Hirano T., Kodama S., Kadowaki Y., Moriyama M., Kawano T., Suzuki M. (2017). Monophosphoryl Lipid A Enhances Nontypeable *Haemophilus influenzae*-Specific Mucosal and Systemic Immune Responses by Intranasal Immunization. Int. J. Pediatr. Otorhinolaryngol..

[23] Ichinohe T., Watanabe I., Ito S., Fujii H., Moriyama M., Tamura S., Takahashi H., Sawa H., Chiba J., Kurata T. (2005). Synthetic Double-Stranded RNA Poly(I:C) Combined with Mucosal Vaccine Protects against Influenza Virus Infection. J. Virol..

[24] Takaki H., Kure S., Oshiumi H., Sakoda Y., Suzuki T., Ainai A., Hasegawa H., Matsumoto M., Seya T. (2018). Toll-like Receptor 3 in Nasal CD103+ Dendritic Cells Is Involved in Immunoglobulin A Production. Mucosal Immunol..

[25] Joseph A. (2002). Liposomal Immunostimulatory DNA Sequence (ISS-ODN): An Efficient Parenteral and Mucosal Adjuvant for Influenza and Hepatitis B Vaccines. Vaccine.

[26] Wang B.-Z., Xu R., Quan F.-S., Kang S.-M., Wang L., Compans R.W. (2010). Intranasal Immunization with Influenza VLPs Incorporating Membrane-Anchored Flagellin Induces Strong Heterosubtypic Protection. PLoS ONE.

[27] Mojgani N., Shahali Y., Dadar M. (2020). Immune Modulatory Capacity of Probiotic Lactic Acid Bacteria and Applications in Vaccine Development. Benef. Microbes.

[28] Villena J., Li C., Vizoso-Pinto M.G., Sacur J., Ren L., Kitazawa H. (2021). *Lactiplantibacillus plantarum* as a Potential Adjuvant and Delivery System for the Development of SARS-CoV-2 Oral Vaccines. Microorganisms.

[29] Medina M., Villena J., Vintiñi E., Hebert E.M., Raya R., Alvarez S. (2008). Nasal Immunization with *Lactococcus lactis* Expressing the Pneumococcal Protective Protein A Induces Protective Immunity in Mice. Infect. Immun..

[30] Villena J., Medina M., Racedo S., Alvarez S. (2010). Resistance of Young Mice to Pneumococcal Infection Can Be Improved by Oral Vaccination with Recombinant *Lactococcus lactis*. J. Microbiol. Immunol. Infect..

[31] Nicod L.P., Cochand L., Dreher D. (2000). Antigen Presentation in the Lung: Dendritic Cells and Macrophages. Sarcoidosis Vasc. Diffus. Lung Dis. Off. J. WASOG.

[32] Kawasaki T., Ikegawa M., Kawai T. (2022). Antigen Presentation in the Lung. Front. Immunol..

[33] Ina Y., Takada K., Yamamoto M., Morishita M., Yoshikawa K. (1991). Antigen-Presenting Capacity of Alveolar Macrophages and Monocytes in Pulmonary Tuberculosis. Eur. Respir. J..

[34] Vecchiarelli A., Dottorini M., Pietrella D., Monari C., Retini C., Todisco T., Bistoni F. (1994). Role of Human Alveolar Macrophages as Antigen-Presenting Cells in *Cryptococcus neoformans* Infection. Am. J. Respir. Cell. Mol. Biol..

[35] Hussell T., Bell T.J. (2014). Alveolar Macrophages: Plasticity in a Tissue-Specific Context. Nat. Rev. Immunol..

[36] Desch A.N., Randolph G.J., Murphy K., Gautier E.L., Kedl R.M., Lahoud M.H., Caminschi I., Shortman K., Henson P.M., Jakubzick C.V. (2011). CD103+ Pulmonary Dendritic Cells Preferentially Acquire and Present Apoptotic Cell–Associated Antigen. J. Exp. Med..

[37] Archambaud C., Salcedo S.P., Lelouard H., Devilard E., de Bovis B., Van Rooijen N., Gorvel J.-P., Malissen B. (2010). Contrasting Roles of Macrophages and Dendritic Cells in Controlling Initial Pulmonary Brucella Infection. Eur. J. Immunol..

[38] Claassen E., Thepen T., Hoeben K., Brevé J., Kraal G. (1993). Migration of Alveolar Macrophages from Alveolar Space to Paracortical T Cell Area of the Draining Lymph Node. Dendritic Cells Fundam. Clin. Immunol..

[39] Kirby A.C., Coles M.C., Kaye P.M. (2009). Alveolar Macrophages Transport Pathogens to Lung Draining Lymph Nodes. J. Immunol..

[40] Kawasaki T., Ikegawa M., Yunoki K., Otani H., Ori D., Ishii K.J., Kuroda E., Takamura S., Kitabatake M., Ito T. (2022). Alveolar Macrophages Instruct CD8+ T Cell Expansion by Antigen Cross-Presentation in Lung. Cell Rep..

[41] Joffre O.P., Segura E., Savina A., Amigorena S. (2012). Cross-Presentation by Dendritic Cells. Nat. Rev. Immunol..

[42] Kopf M., Schneider C., Nobs S.P. (2015). The Development and Function of Lung-Resident Macrophages and Dendritic Cells. Nat. Immunol..

[43] Boyaka P.N., Fujihashi K. (2019). Host Defenses at Mucosal Surfaces. Clinical Immunology.

[44] Fujihashi K., Sato S., Kiyono H. (2014). Mucosal Adjuvants for Vaccines to Control Upper Respiratory Infections in the Elderly. Exp. Gerontol..

[45] Janoff E.N., Fasching C., Orenstein J.M., Rubins J.B., Opstad N.L., Dalmasso A.P. (1999). Killing of *Streptococcus pneumoniae* by Capsular Polysaccharide–Specific Polymeric IgA, Complement, and Phagocytes. J. Clin. Investig..

[46] Sun K., Johansen F.-E., Eckmann L., Metzger D.W. (2004). An Important Role for Polymeric Ig Receptor-Mediated Transport of IgA in Protection against *Streptococcus pneumoniae* Nasopharyngeal Carriage. J. Immunol..

[47] Park S.-M., Ko H.-J., Shim D.-H., Yang J.-Y., Park Y.-H., Curtiss R., Kweon M.-N. (2008). MyD88 Signaling Is Not Essential for Induction of Antigen-Specific B Cell Responses but Is Indispensable for Protection against *Streptococcus pneumoniae* Infection Following Oral Vaccination with Attenuated Salmonella Expressing PspA Antigen. J. Immunol..

[48] Ferreira D.M., Darrieux M., Silva D.A., Leite L.C.C., Ferreira J.M.C., Ho P.L., Miyaji E.N., Oliveira M.L.S. (2009). Characterization of Protective Mucosal and Systemic Immune Responses Elicited by Pneumococcal Surface Protein PspA and PspC Nasal Vaccines against a Respiratory Pneumococcal Challenge in Mice. Clin. Vaccine Immunol..

[49] Fukuyama Y., King J.D., Kataoka K., Kobayashi R., Gilbert R.S., Oishi K., Hollingshead S.K., Briles D.E., Fujihashi K. (2010). Secretory-IgA Antibodies Play an Important Role in the Immunity to *Streptococcus pneumoniae*. J. Immunol..

[50] Miyasaka T., Akahori Y., Toyama M., Miyamura N., Ishii K., Saijo S., Iwakura Y., Kinjo Y., Miyazaki Y., Oishi K. (2013). Dectin-2-Dependent NKT Cell Activation and Serotype-Specific Antibody Production in Mice Immunized with Pneumococcal Polysaccharide Vaccine. PLoS ONE.

[51] Saeland E., Vidarsson G., Leusen J.H., Van Garderen E., Nahm M.H., Vile-Weekhout H., Walraven V., Stemerding A.M., Verbeek J.S., Rijkers G.T. (2003). Central Role of Complement in Passive Protection by Human IgG1 and IgG2 Anti-Pneumococcal Antibodies in Mice. J. Immunol..

[52] Bittar F., Cassagne C., Bosdure E., Stremler N., Dubus J.-C., Sarles J., Reynaud-Gaubert M., Raoult D., Rolain J.-M. (2010). Outbreak of *Corynebacterium pseudodiphtheriticum* Infection in Cystic Fibrosis Patients, France. Emerg. Infect. Dis..

[53] Van Roeden S.E., Thijsen S.F., Sankatsing S.U.C., Limonard G.J.M. (2015). Clinical Relevance of *Corynebacterium pseudodiphtheriticum* in Lower Respiratory Tract Specimens. Infect. Dis..

[54] Ogawa Y., Ote H., Arai T., Kazama R., Kimura K., Nagata T., Kumasawa J., Kohno M., Kohata H., Nishida K. (2022). *Corynebacterium pseudodiphtheriticum* as a Pathogen in Bacterial Co-Infection in COVID-19 Patients on Mechanical Ventilation. Jpn. J. Infect. Dis..

[55] Wang Y., Hosomi K., Shimoyama A., Yoshii K., Nagatake T., Fujimoto Y., Kiyono H., Fukase K., Kunisawa J. (2021). Lipopolysaccharide Derived From the Lymphoid-Resident Commensal Bacteria *Alcaligenes faecalis* Functions as an Effective Nasal Adjuvant to Augment IgA Antibody and Th17 Cell Responses. Front. Immunol..

